# Magnitude and risk factors for hypertension among public servants in Tigray, Ethiopia: A cross-sectional study

**DOI:** 10.1371/journal.pone.0204879

**Published:** 2018-10-03

**Authors:** Alemayehu Bayray, Kidanu Gebremariam Meles, Yosef Sibhatu

**Affiliations:** School of Public Health, College of Health Sciences, Mekelle University, Mekelle, Ethiopia; National Institute of Health, ITALY

## Abstract

**Background:**

Hypertension is a globally recognized threat to social and economic development with premature morbidity and mortality. In middle and low-income countries hypertension appears to be increasing. However, sufficient data on this silent-killer is not available in Ethiopia. Therefore, this study examined the magnitude and risk factors for hypertension among public servants in Tigray, Ethiopia.

**Methods:**

We used a cross-sectional survey from May-June 2016 among 1525 public servants in Tigray region. Field workers collected data using a pre-tested, standardized questionnaire. A multivariate logistic regression analysis conducted to identify risk factors for hypertension. Statistical significance was declared using a p-value<0.05 and 95% of confidence interval (CI) for an adjusted odds ratio (AOR).

**Results:**

The overall prevalence of hypertension was 16% (95% CI: 13.10–21.9) and the proportion of awareness (96.7%), treatment (31.3%) and control of hypertension (40.1%) among employees. Being male [AOR = 2.06, 95%CI:1.49, 2.84], ages groups of 30–49 years [AOR = 2.21, 95%CI:1.25, 3.89] and >50years [AOR = 3.61, 95% CI:1.93, 6.69], Body Mass Index(BMI); underweight [AOR = 0.40, 95% CI; 0.20, 0.78], overweight [AOR = 1.70, 95%CI; 1.22, 2.33] and obesity [AOR = 3.20, 95% CI; 1.78, 5.78] were determinants for hypertension.

**Conclusion:**

The prevalence of hypertension is relatively high in Mekelle city compared with previous reports. This study revealed that male sex, age-group, and BMI were evidenced as risk factors for hypertension. Policy makers need to consider sector wise integrating prevention and control of hypertension. Skilled based information, education and communication strategies should be designed and implemented to avoid unhealthy lifestyles, investing in workforces to eliminate the modifiable risk factors for non-communicable diseases and promote healthy practices.

## Introduction

Hypertension is the most common single risk factor for cardiovascular related deaths and disability globally [1]. Hypertension is a worldwide silent public-health challenge and a leading modifiable risk factor for non-communicable disease (NCD) and mortality. In 2015 and 2016 W.H.O. report shows that 40 million deaths and more than 70% of mortality worldwide had been caused by NCDs respectively. More than 40% of these deaths were premature and 80% of these deaths from NCDs occur in low and middle-income countries [2]. Hypertension is one of the most frequently observed risk factor for cardiovascular disease (CVD) in Sub-Saharan African [3]. In Sub-Saharan Africa, countries are experiencing an unexpected rise in the incidence of hypertension [1].

By 2030, the number of deaths from NCDs worldwide is projected to increase to 52 million annually [2]. The few studies conducted in Ethiopia are showing a high prevalence of the disease in the country. Approximately, 10.5% of the Ethiopian population and 30% of adults in Addis Ababa has been estimated to have hypertension [4,5]. Another study in Ethiopia showed the overall prevalence of hypertension among adult population to be 27.9%, with the proportion being 30.7% in urban and 25.3% in rural residents. In terms of sex proportion, 29.3% was for women and 26.3% for men [6].

According to WHO 2011 report, the Age-standardized death rate of cardiovascular diseases and hypertension in Ethiopia was estimated to be 473 per 100,000 populations, much higher than that in Japan [7]. Cumulative economic losses associated with the most common NCDs like hypertension, DM in low and middle-income countries are projected to be over $7 trillion during the years 2011–2025, pushing millions of people below the poverty line [8]. Ethiopia has not yet thoroughly studied the epidemiological situation of hypertension nor did they establish systematic programs for prevention and control at a workplace where a sedentary lifestyle and working environment exposes workers more to develop hypertension. Therefore, this study aimed to investigate the magnitude and risk factors for hypertension among public servants in the Tigray Region.

## Methods and materials

### Study design, area and period

We used a cross-sectional survey to assess the prevalence and risk factors of hypertension among public servants in Tigray from May-August, 2016. Tigray is found in the northern part of Ethiopia. Mekelle is its capital city. Based on the 2007 Census conducted by the Central Statistical Agency of Ethiopia (CSA), the Region has a projected population of 5,151,998, of whom 2,539,997 are male and urban inhabitants 1,331,000. There are more than 20 public offices in the city where an estimated 30,000 public servants are working until the retirement age of 65 years.

### Source and study of population

We sampled all adult public servants aged 18–64 years residing in Mekelle city. We excluded pregnant and breastfeeding women from participation in the study.

### Sample size determination and sampling procedure

We selected respondents by a random sampling technique stratified by offices based on employee registration records. Purposive (convenient) samplings on a voluntary basis were applied, when randomly selected subjects are not accessible.

### Data collection technique and tool

Field workers collected data using a standardized, structured and pre-tested questionnaire [9]. Initially, the questionnaire was prepared in English and translated into Tigrigna (local language). Three trained supervisors and 10 data collectors participated in the data collection process. All of the data collectors had data collection experience in a health survey.

### Measurements

We used a standardized questionnaire adapted from the World Health Organization STEPS instrument developed for use in resource-limited countries [9] to collect data on socio-economic characteristics, history of infectious and chronic disease diagnoses, and common risk factors for hypertension including tobacco use, alcohol use, and physical activity. Physical measurements and biochemical measurements were made following a standardized protocol across the study sites. Physical measurements included weight, height and blood pressure.

Blood pressure measurements (BP) were taken on the left arm with the participant in the sitting position using an Arm-Omron digital blood pressure monitor device. The blood pressure of subjects was measured on two different occasions at least 5 minutes apart and the average of the last two blood pressure readings was used for all the subjects. The participants were considered to be hypertensive if they had a systolic blood pressure of ≥140 mmHg and/or a diastolic blood pressure of ≥90 mmHg [10].

Body weight was measured using a bathroom weighing scale that was calibrated for each patient. Subjects were weighed bare-footed in light clothing, the same weighing scale was used for all the subjects and readings expressed in kilograms (kg) to the nearest 0.5kg. Height was also measured with the subjects standing upright, barefooted, back and heel against the wall facing forward using a collapsible standard metal ruler mounted against the wall, while the height was read by placing a ruler at the highest point of the subject’s scalp and expressed to the nearest centimeters. The body mass index (BMI) (kg/m2) was used to define obesity and classified as follows: Underweight = BMI <18.5, Normal weight = BMI 18.5–24.9, Overweight = BMI 25.0–29.9 and Obesity = BMI ≥30.0 [11]. The biochemical measurements were total cholesterol level, HDL cholesterol, LDL cholesterol, and triglycerides. We collected a small amount of peripheral blood sample from the participants and tested it on-spot immediately after the collection.

### Data quality assurance

We used a pre-tested structured and standardized questionnaire adopted from WHO STEP [9].

The questionnaire was initially prepared in English[S1 file] and translated into Tigrigna, the local language by a language expert. We gave two days training for data collectors and supervisors. We conducted a pre-test on five percent of the total sample size before the actual data collection.

### Data processing and analysis

Data-clerks entered the data into Epi-Info version7.1.5(Center for Disease Control and Prevention, USA) and analyzed using SPSS version 20.0(SPSS Chicago, IL, USA). Bivariate and multivariate logistic regression analysis conducted to assess risk factors for hypertension among public servants at a P-value<0.05, 95% confidence interval and odds ratio.

### Ethical considerations

We obtained informed written consent from participants. Data collection was conducted confidentially and data de-identified, de-linked and stored in a secure location. Informed verbal consent was obtained from each subject so that the study could be published and presented at different workshops while protecting the participants’ confidentiality. The informed verbal consent procedure was specifically approved this study by the Institutional Review Board of Mekelle University College of Health Sciences.

## Results

### Socio-demographic characteristics of respondents

In this study, a total of 1523 respondents were involved, making a response rate of 99.7%. More than half of the respondents, 871(57.2%) were females. The majority of the respondents (65.4%) were in the age group of 30-49years. More than three-fourths, 1451(95.3%) were Orthodox and 953(62.6%) were married. More than 50%, 868(57.0%) respondents had completed college/university education and 823(54.0%) earned of 118.59$US (**Table 1).**

**Table 1:** Socio-demographic characteristics of the public servants Tigray, northern Ethiopia, 2016, (n = 1523).

Characteristics		Frequency	Percentage
** Sex**	Male	871	57.2
	Female	652	42.8
**Age group (n = 1500)**	18–29	288	19.2
	30–49	981	65.4
	≥50	231	15.4
** Religio**n	Orthodox-Christians	1451	95.3
	Others*	72	4.7
** Marital status**	Never married	436	28.6
	Currently married	953	62.6
	Divorced/ Widowed	134	8.8
** Educational status**	Write and read only	71	4.7
	Primary education (1–8)	60	3.9
	Secondary education (9–12)	151	9.9
	College and above	868	57.0
	Master's degree holders	373	24.5
** Monthly income (USD)**	<35.89	114	7.5
	35.90–80.85	330	21.7
	80.86–119.5 8	256	16.8
	≥118.59	823	54.0

Others

* Catholic, Protestant and Muslim

### Behavioral, dietary and bio-medical related characteristics

More than three-fourths of 1134(85.4%) respondents consumed alcohol in the last 30 days and 26(1.7%) ever chat chewier. More than half, 784(51.5%) of respondents reported that they consumed fruits. Roughly, more than two-thirds, 1175(77.5%) of respondents stated that they consumed vegetables and only 52(4.5%) consumed three days or more per week. Almost all 1474(97.2%) respondents were physically inactive. Approximately, one fourth 364(24.3%) of the respondents were overweight and 61(4.1%) obese. Regarding dyslipidemia 385(25.1%) of respondents had raised total cholesterol (> = 200mg/dl), 874(57.3%) raised triglyceride (> = 150mg/dl), 448(29.4%) > = 130mg/dl LDL and 470(30.8%) high HDL cholesterol (**Table 2**).

**Table 2:** Behavioral, dietary and bio-medical related characteristics of public servant in Tigray, northern Ethiopia, 2016, (n = 1523).

Characteristic		Frequency	Percentage
**Currently Smoking**	Yes	30	2.0
	No	1493	98.0
**Alcohol use in the last 30 days (n =** 1328)	Yes	1134	85.4
	No	194	14.6
**Frequency of alcohol use (n =** 1339)	Daily	41	3.1
	Twice per week	366	27.3
	1–3 times per month	932	69.6
**Chat chewing**	Yes	26	1.7
	No	1497	98.3
**Consume fruit**	Yes	784	51.5
	No	739	48.5
**Fruit consumption per week**	<3 days	747	96.0
	≥3 days	31	4.0
**Consume vegetable**	Yes	1175	77.5
	No	342	22.5
**Vegetable consumption per week**	<3 days	1109	95.5
	≥3 days	52	4.5
**Spicy foods used**	Yes	79	5.2
	No	1443	94.8
**Physical activity**	Active	43	2.8
	Inactive	1474	97.2
**Body Mass Index (BMI, kg/m**^**2**^)	Normal	852	56.9
	Underweight	220	14.7
	Overweight	364	24.3
	Obese	61	4.1
**Ever measured cholesterol level**	Yes	64	4.2
	No	1448	95.8
**Raised total cholesterol**	**<200mg/dl**	1142	74.9
	**≥200mg/dl**	385	25.1
**Raised triglyceride**	**<150mg/dl**	651	42.7
	**≥150mg/dl**	874	57.3
**LDL**	**<130**	1077	70.6
	**≥130**	448	29.4
HDL cholesterol**	Lowl Low	1053	69.2
	High	470	30.8

HDL cholesterol

**: High for men > = 40 and for women > = 50

### Prevalence, awareness, treatment, and control of hypertension

The overall hypertension proportion was 16% (95%CI:13.10, 21.9). Among all hypertensive employees identified, almost all (96.7%) were aware of their hypertension status. However, nearly half, 109(44.9%) of respondents had ever been notified their blood pressure were raised. More than 30% of respondents were taking an anti-hypertensive drug and the overall control rate of hypertension was 40.1% (**Table 3).**

**Table 3:** Prevalence, awareness, treatment, and control of hypertension among public servant in Tigray, northern Ethiopia, 2016, (n = 1523).

Characteristics	Frequency	Percentage
Currently hypertensive (n = 1523)	243	16.0
Ever measured blood pressure (n = 243)	235	96.7
Ever been told by a health professionals that they have hypertension (n = 243)	109	44.9
Have taken anti-hypertensive medication (n = 243)	76	31.3
Hypertension controlled (n = 76)	35	40.1

### Factors associated with hypertension among public servants

Sex, age group, and BMI status (underweight, overweight and obesity) were identified as determinants for hypertension among public servants declared with odds ratio and 95% CI in multivariate logistic regression.

The likelihood of being hypertensive was two times more likely higher among male [AOR = 2.06, 95%CI: 1.49, 2.84] compared to their counterparts. Employees in the age groups of 30–49 years [AOR = 2.20, 95%CI: 1.31, 5.07] and ≥50 years [AOR = 3.61, 95%CI: 1.9, 6.69] were two to almost four folds more likely to develop hypertension than those in the age group of 18–29 years.

Moreover, overweight respondents were almost two times [AOR = 1.69, 95%CI:1.22, 2.33] more likely to be hypertensive than participants with normal BMI. Obese respondents were more than three times [AOR = 3.21, 95%CI; 1.78, 5.78] at increased risk of being hypertensive compared with respondents with normal BMI. Whereas underweight participants were 60% less likely [AOR = 0.40, 95% CI; 0.20, 0.78] to develop hypertension (**Table 4**).

**Table 4:** Logistic regression analyses of associated factors with hypertension among public servant, Tigray, northern Ethiopia, 2016, (n = 1523).

Variables	Hypertension status		COR (95%CI)	AOR (95%CI)
	Yes (%)	No (%)		
**Sex**				
** Male**	175(72.0)	696(54.4)	2.16(1.59, 2.92)	2.06(1.49, 2.84)
** Female**	68(28.0)	584(45.6)	**1**	**1**
**Age group (years)**				
** 18–29**	15(6.3)	273(21.6)	**1**	**1**
** 30–49**	163(68.2)	818(64.9)	3.63(2.10, 6.26)	2.20(1.25, 3.89)
** ≥50**	61(25.5)	170(13.5)	6.53(3.59, 11.86)	3.61(1.93,6.69)
**Educational status**				
** Write and read only**	9(3.7)	61(4.8)	0.66(0.31, 1.40)	-
** Primary education (1–8)**	13(5.4)	46(3.6)	1.26(0.65, 2.47)	-
** Secondary education (9–12)**	23(9.5)	12(9.9)	0.86(0.52, 1.44)	-
** College and above**	130(53.7)	738(57.8)	0.81(0.58, 1.11)	-
** Master's degree holders**	67(27.7)	305(23.9)	**1**	
**Monthly income (US Dollar)**				
** <35.89**	7(2.9)	107(8.4)	0.29(0.13, 0.63)	-
** 35.90–80.85**	37(15.2)	293(22.9)	0.55(0.38, 0.81)	-
** 80.86–119.5 8**	46(18.9)	210(16.4)	0.96(0.67, 1.38)	-
** ≥118.59**	153(63.0)	670(52.3)	1	1
**Alcohol used in the last 30 days**				
** Yes**	199(91.7)	935(84.2)	2.08(1.25, 3.46)	-
** No**	18(8.3)	176(15.8)	1	1
**Total cholesterol**				
** <200mg/dl**	164(67.5)	978(76.4)	1	1
** ≥200mg/dl**	79(32.5)	302(23.6)	1.56(1.16,2.10)	-
**Triglyceride**				
** <150mg/dl**	85(35)	566(44.2)	1	1
** ≥150mg/dl**	158(65)	714(55.8)	1.47(1.11,1.96)	-
**LDL**				
** <130**	155(63.8)	922(72)	1	1
** ≥130**	88(36.2)	358(28)	1.46(1.10,1.95)	-
**HDL cholesterol**				
** Low**	179(73.7)	874(68.3)	1	1
** High**	64(26.3)	406(31.7)	0.77(0.57, 1.05)	-
**BMI (kg/m** ^**2**^ **)**				
** Normal**	123(51)	729(58)	1	1
** Underweight**	210(16.7)	10(4.1)	0.28(0.15,0.55)	0.4(0.20,0.78)
** Overweight**	86(35.7)	278(22.1)	1.83(1.35, 2.49)	1.70(1.22, 2.33)
** Obesity**	22(9.1)	39(3.1)	3.34(1.92, 5.83)	3.20(1.78, 5.78)

COR = Crude Odds Ratio, AOR = Adjusted Odds Ratio

## Discussion

This study showed that the proportion of hypertension among public servants to be 16%. Sex, age and BMI status (underweight, overweight and obesity) were determinants for hypertension among public employees.

The prevalence of hypertension was consistent with studies done in southwest Ethiopia (13.2%) [12], Northern Ethiopia (18.1%) [13] and in Aksum town (16.5%) [14]. However, this prevalence is lower than that of studies evidenced in Addis Ababa, Ethiopia (27.3%) [15], Angola (23%) [5] and Kenya (30%) [4]. The observed discrepancies among studies including the present study could be due to study population characteristics and settings difference; the current study included public servants as study subjects whereas almost all the comparative studies were general population.

The study identified the risk factors for hypertension; male employees were two times more likely to develop hypertension compared to female employees. Similar findings have been reported across the low-income countries; a study investigated an adult population in Southern Ethiopia [16] revealed that being a male was a risk factor for hypertension; similarly in young adult Ugandan population [17] and in Brazil men were 49% more likely to have hypertension than women [18]. In the Ethiopian context, men are proxy for common major modified risk factors for tobacco use, alcohol consumption and khat chewing and this is well evidenced in STEPs Survey on risk factors for NCDs and prevalence of selected NCDs, Ethiopia [19]. This could also be due to the high proportion of male employees with hypertension (72%) in the present study.

Employees whose age groups were from 39–49 years and ≥ 50-years were three to nearly five times more likely to have hypertension compared to young age group. This finding is in line with community-based studies conducted in Gondar [20], Bedele [21] and Durame town [16], Ethiopia. Similarly, age has been evidenced as the main predictor of hypertension in low-income countries’ settings such as the Ugandan population [17]. As ages of individuals increase; they could probably be contracted by non-communicable diseases including hypertension. This is reported in studies conducted in a hospital-based and community-based, southwest Ethiopia [12,16,21].

Furthermore, BMI was evidenced as a risk factor for hypertension; those employees who were categorized under over-weight and obesity were more likely to be hypertensive compared to their normal counterparts. This result is consistent with studies conducted in different settings of Ethiopia; in Aksum [14], Gondar [22] and Durame town [16]. A study conducted on adult young Ugandan population has shown that obesity was associated with hypertension [17] and other study conducted in three populations in Africa and Asia (Vietnamese, Indonesian and Ethiopian) also revealed that a significant positive correlation between BMI and blood pressure was also observed [23]. Body mass index (BMI) is usually noted as proxy and mediator factor for hypertension; the observed relationship between overweight, obesity, and hypertension could be due to the difference in dietary habits and physical inactivity of study participants. In this study, significant proportions of public employees were classified under unhealthy diets such as low fruit and vegetable consumption per week and physically inactive. This implies that those individuals who are labeled as overweight or obesity are exposed to major non-communicable diseases. However, underweight was found to be a protective factor for hypertension. This could be because the nutritional status of study participants was measured using BMI which indicates protein energy malnutrition which in turn is explained by underweighted adults being low risks for metabolic syndrome (dyslipidemia) such as raised total cholesterol, triglyceride, LDL and HDL.

### Strength and limitation of the study

Data were collected using a pretested and standardized tool (WHO steps). We measured biochemical and physical measurements using digitalized devices. Measurement error (misclassification) was not also observed in this study. This cross sectional study was conducted in urban setting among public servants that did not show the actual burden of hypertension in both rural and urban setting among general populations and was identified as limitation.

## Conclusion

The proportion of hypertension is relatively high among public servants in Mekelle city and the figure shows an increase compared with previous reports. This study revealed that sex, age, and BMI were the risk factors for hypertension in the public servants. Policy makers need to consider sector-wise integrated prevention and control measures of hypertension. Appropriate information, education and communication strategies should be designed and implemented to avoid unhealthy lifestyles, investing in workforces to eliminate the modifiable risk factors for hypertension and promote healthy practices. Further research on health seeking behaviors toward hypertension treatment and prevention mechanisms is important so as to formulate possible strategies.

## Supporting information

S1 File English version STEPS_Instrument_V3.1.pdf, 2016. (PDF)
