# Expression of Concern: Magnitude and risk factors for hypertension among public servants in Tigray, Ethiopia: A cross-sectional study

**DOI:** 10.1371/journal.pone.0279946

**Published:** 2023-01-05

**Authors:** The PLOS ONE Editors

After publication of this article [1], concerns were raised about the reporting of the methodology, the description and ownership of the dataset used in this study, and the relationship of this article to a previous cross-sectional survey of non-communicable disease risk factors (including hypertension) that sampled a similar population [2]. The *PLOS ONE* Editors have been unable to contact the authors or Mekelle University to discuss these issues. However, we understand that substantial communication issues have been affecting the region for an extended period.

A statistical advisor reviewed the article [1] and advised that the reporting of the methodological details is incomplete, and that additional information is needed to clarify the sampling and measurement methods, and how the biomedical information was collected. The statistical advisor also noted that the article does not clearly report what variables are included in the logistic regression models, and that no information on collinearity checks is provided. They advised that since the model used is incomplete and not sufficiently adjusted, the conclusion regarding magnitude should be interpreted with caution. It was also noted that the study reported conclusions as to risk factors for hypertension, but as this was a cross-sectional, observational study, conclusions about potential causal relationships are not supported.

In light of the unresolved questions about the dataset and methodology, the *PLOS ONE* Editors issue this Expression of Concern.

At the time of this notice, the article [1] was republished to address an issue with a Supporting Information file.

Note: in 2019, the authors posted an update to the Acknowledgments and Funding Statement in a Comment on the article’s [1] *PLOS ONE* webpage.
